# Implementing Behavioral Health Social Workers' Increased Mental Health Care Among Older Adults

**DOI:** 10.1093/geroni/igab046.2077

**Published:** 2021-12-17

**Authors:** Elizabeth Pfoh, Jessica Hohman, Kathleen Alcorn, Nirav Vakharia, Michael Rothberg

**Affiliations:** Cleveland Clinic, Cleveland, Ohio, United States

## Abstract

Depression is underdiagnosed and undertreated among older adults. Health systems can screen patients to identify depression, but systemic linkages to treatment are required to ensure care. We used a retrospective stepped-wedge study to identify the impact of implementing behavioral health social workers (BHSWs) on receipt of treatment after a new depression diagnosis. We included adults over 65 years of age with a primary care visit between 2016 and 2019 at a large integrated health system. We excluded patients who were diagnosed with or treated for depression in 2015. Patients were categorized into control (diagnosed before implementation) and intervention (diagnosed after implementation) groups. From our electronic health record, we collected prescriptions for pharmacotherapy and behavioral health visits. Patients were considered treated if they received pharmacotherapy or had a behavioral health visit within 30 days of diagnosis. We used multilevel logistic regression models to identify the association between implementation period (pre versus post) and treatment, adjusted for demographic variables and clustering within site. Our population included 4,475 people. The percent of patients that received treatment increased from 47% to 54% after implementation and the percent of patients with ≥1 behavioral health visit within 30 days increased from 3% to 8% (p<0.01, respectively). The adjusted odds ratio of receiving treatment (AOR: 4.13, 95%CI: 2.84-6.01) and having a behavioral health visit (AOR: 3.12, 95%CI: 2.31-4.24) was significantly higher in the post-implementation period. In conclusion, implementation of BHSWs was associated with increased treatment for older patients with depression.

